# Recombination between the mouse Y chromosome short arm and an additional Y short arm-derived chromosomal segment attached distal to the X chromosome PAR

**DOI:** 10.1007/s00412-015-0559-0

**Published:** 2015-11-23

**Authors:** Fanny Decarpentrie, Obah A. Ojarikre, Michael J. Mitchell, Paul S. Burgoyne

**Affiliations:** Department of Stem Cell Biology and Developmental Genetics, MRC National Institute for Medical Research, The Ridgeway, Mill Hill, London, NW7 1AA UK; Mill Hill Laboratory, The Francis Crick Institute, London, NW7 1AA UK; Inserm UMR_S 910, Aix Marseille Université, 13005 Marseille, France

**Keywords:** Mouse, Meiosis, Y-chromosome, Short arm (Yp), Recombination, *Spo11*

## Abstract

In a male mouse, meiosis markers of processed DNA double strand breaks (DSBs) such as DMC1 and RAD51 are regularly seen in the non-PAR region of the X chromosome; these disappear late in prophase prior to entry into the first meiotic metaphase. Marker evidence for DSBs occurring in the non-PAR region of the Y chromosome is limited. Nevertheless, historically it has been documented that recombination can occur within the mouse Y short arm (Yp) when an additional Yp segment is attached distal to the X and/or the Y pseudoautosomal region (PAR). A number of recombinants identified among offsprings involved unequal exchanges involving repeated DNA segments; however, equal exchanges will have frequently been missed because of the paucity of markers to differentiate between the two Yp segments. Here, we discuss this historical data and present extensive additional data obtained for two mouse models with Yp additions to the X PAR. PCR genotyping enabled identification of a wider range of potential recombinants; the proportions of Yp exchanges identified among the recombinants were 9.7 and 22.4 %. The frequency of these exchanges suggests that the Yp segment attached to the X PAR is subject to the elevated level of recombinational DSBs that characterizes the PAR.

**Electronic supplementary material:**

The online version of this article (doi:10.1007/s00412-015-0559-0) contains supplementary material, which is available to authorized users.

## Introduction

In a normal male mouse meiosis, the regular *Spo11*-dependent recombination that occurs between the X and Y chromosomes is efficiently restricted to their PAR regions (Burgoyne 1982; Kauppi et al. 2011), although X-Y synapsis may spread beyond the PAR boundary during the mid pachytene stage. The initial synapsis of the PARs protects the PAR chromatin from meiotic transcriptional silencing that is initiated at the zygotene/pachytene transition (reviewed by Burgoyne et al. 2009), but it is not widely appreciated that the subsequent spreading of synapsis may well interfere with the transcriptional silencing of X-Y chromatin proximal to the PARs. This is likely the explanation for the occasional finding of pachytene spermatocytes transcribing the X-linked gene *Scml2* (Royo et al. 2010), since it is located only ∼7 Mb from the X PAR (total X length ∼177 Mb).

Although immunostaining for proteins that localize to sites of DSBs (e.g. RAD51, DMC1, RPA) have shown that the X has multiple DSBs that could enable recombination with a synapsed non-PAR Y segment lacking DSBs (Kauppi et al. 2011, 2012; Moens et al. 1997; Plug et al. 1998), translocations between the X and the Y are extremely rare. This is presumably because regions of homology are very limited and unlikely to be juxtaposed during the presynaptic homology search. This is supported by the sequence data for multi-copy X and Y paralogs that indicate crossovers leading to exchange between X and Y copies are very rare despite substantial homology (Soh et al. 2014). Markers of DSBs have rarely been reported on the non-PAR Y, but it can participate in homology-driven recombination as evidenced by mouse and human Y chromosome sequence data, which implicate intra-chromatid or inter-sister recombination between ampliconic repeats as responsible for the generation of rare Y deletions and rearrangements (Lange et al. 2009, 2013; Skaletsky et al. 2003; Soh et al. 2014). It is therefore reasonable to posit that rare DSBs in Yp could participate in rare recombination with a *synaptic* partner with substantial homology, provided they are juxtaposed during the presynaptic homology search.

**Fig. 1:**
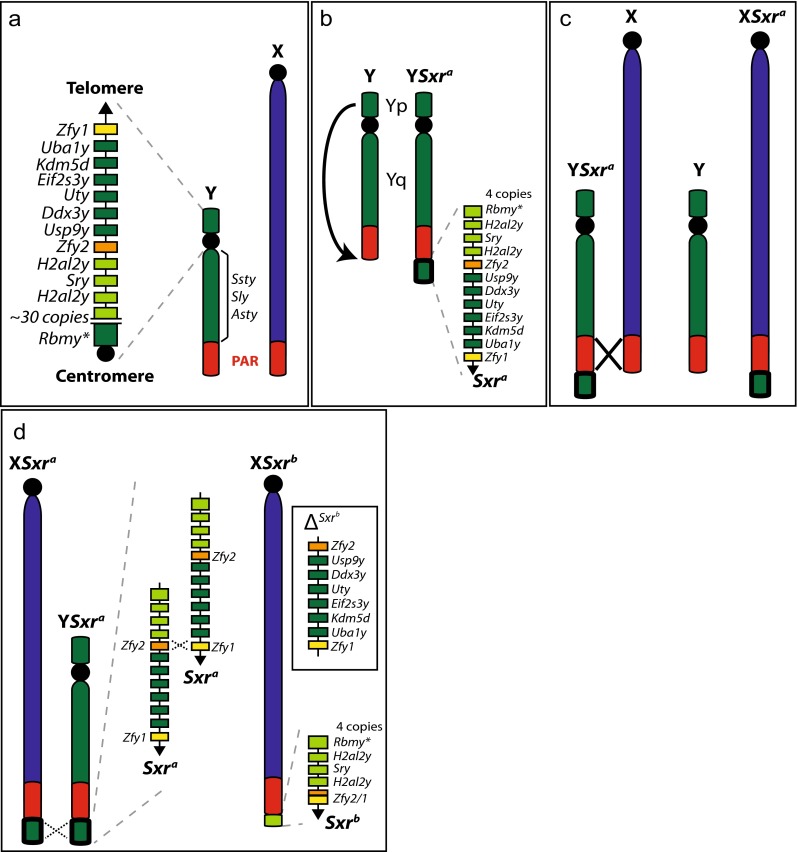
Origin of *Sxr* variants. (**a**) Wild type XY. (**b**) Duplication and translocation of Yp created *Sxr* ^*a*^. (**c**) PAR-PAR recombination in XY*Sxr* ^*a*^ males generates the X*Sxr* ^*a*^ chromosome. (**d**) An unequal crossover in an X*Sxr* ^*a*^Y*Sxr* ^*a*^ male created the *Sxr* ^*b*^ deletion variant. (Color codes: *Red* = PAR; *Blue* = non-PAR X; *Dark green* = non-PAR Y except *Sxr* ^b^; *Light green* = *Sxr* ^*b*^; *Yellow* = *Zfy1*; *Orange* = *Zfy2*; *Orange/Yellow* = *Zfy2/1* fusion gene.) ^*^ *Rbmy* copy number estimates based on information provided by Soh et al. (2014) and Mahadevaiah et al. (1998)

That this may be the case is supported by the historical identification of occasional Y short arm recombinants among the progeny of males in which there was a Y short arm (Yp) derivative (*Sxr*^*a*^ or *Sxr*^*b*^—see Fig. 1b, d) attached distal to the X PAR, or distal to the X and Y PARs (Epplen et al. 1988; Laval et al. 1995; McLaren et al. 1988, 1992; Simpson et al. 1984). While there was the expected frequent transfer of the complete *Sxr* segment from the Y PAR to the X PAR (Fig. 1c) or vice versa, there were also exchanges of partial *Sxr* segments with Yp; the latter exchanges must involve DSBs located within *Sxr* or within Yp. These partial *Sxr* exchanges could be balanced exchanges or unbalanced exchanges; the latter are an expected consequence of the presence of substantial regions of sequence repeats on the mouse Y short arm (Soh et al. 2014).

Two of these studies are particularly relevant in the context of the new data reported here. Firstly, Simpson et al. (1984) identified among the progeny of an X*Sxr*Y*Sxr* male (with *Sxr* attached to the X and Y PARs) a mouse carrying an *Sxr* deletion variant that was designated *Sxr*^*b*^ (with the original *Sxr* then being designated *Sxr*^*a*^). Subsequent work established that the deletion resulted from a crossover located within *Zfy1* of the Y-located *Sxr* and within *Zfy2* of the X-located *Sxr*, resulting in a 1.38-Mb deletion removing six Yp genes and creating a *Zfy2/1* fusion gene (Fig. 1d) (Decarpentrie et al. 2012; Mazeyrat et al. 1998; Simpson and Page 1991). Secondly, McLaren et al. (1992) analyzed the progeny of X*Sxr*^*b*^Y*Sxr*^*a*^ males that enabled them to document exchanges between *Sxr*^*a*^ and *Sxr*^*b*^ and between *Sxr*^*b*^ and Yp. Significantly, the *Sxr*^*a*^-*Sxr*^*b*^ exchanges must have involved DSBs located within these Yp derivatives attached to the PARs. Here, we report a strikingly high frequency of *Sxr*^*b*^-Yp recombination in two mouse models that have *Sxr*^*b*^ attached to the X PAR; we propose that this high frequency of DSBs is a consequence of the spreading of DSB hotspot activity from the X PAR into *Sxr*^*b*^.

## Materials and methods

### Mouse breeding

**Fig. 2:**
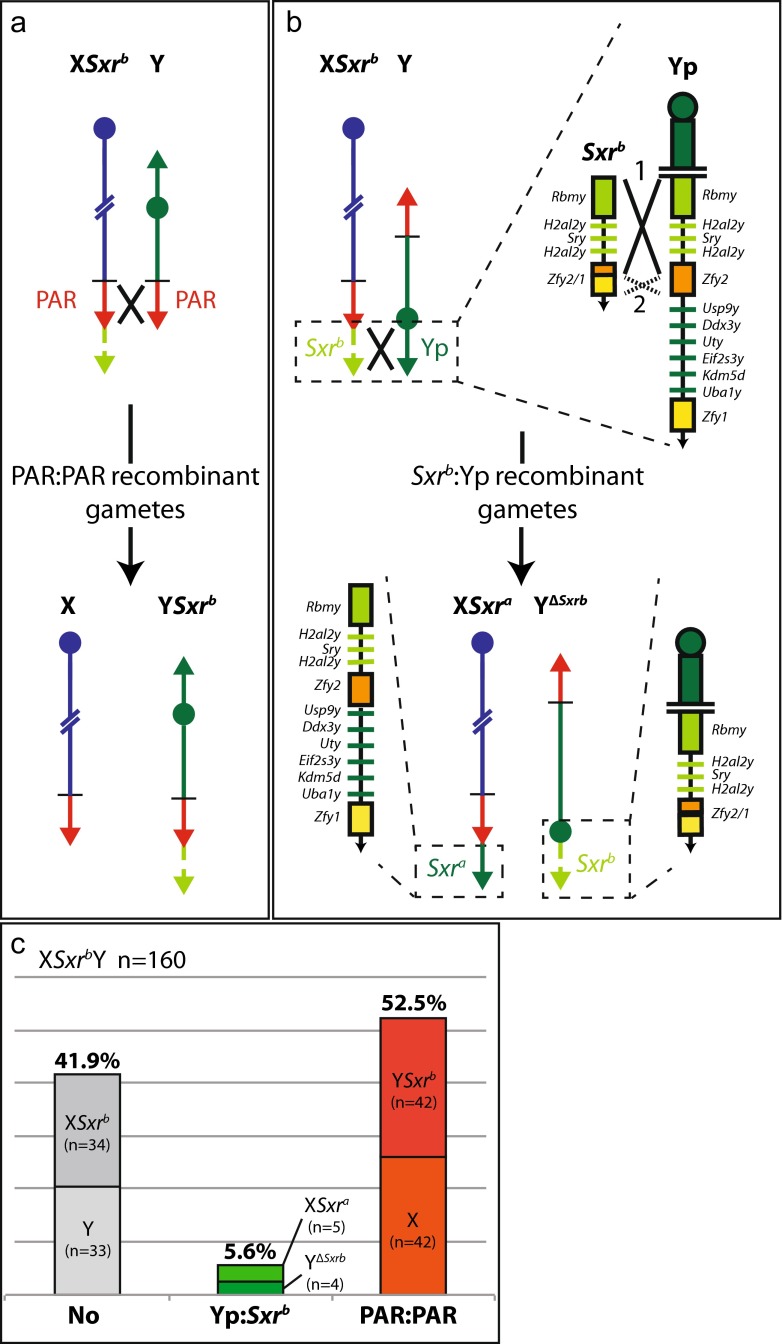
Recombination in X*Sxr* ^*b*^Y males. (**a**) PAR-PAR recombination. (**b**) Yp*-Sxr* ^*b*^ recombination with expanded views showing the Y gene content of the paired segments and of the two types of recombinant from crossover 1 (*black cross*); (**c**) Bar chart of non-recombinant (No) and recombinant (Yp-*Sxr* ^*b*^, PAR-PAR) frequencies

**Fig. 3:**
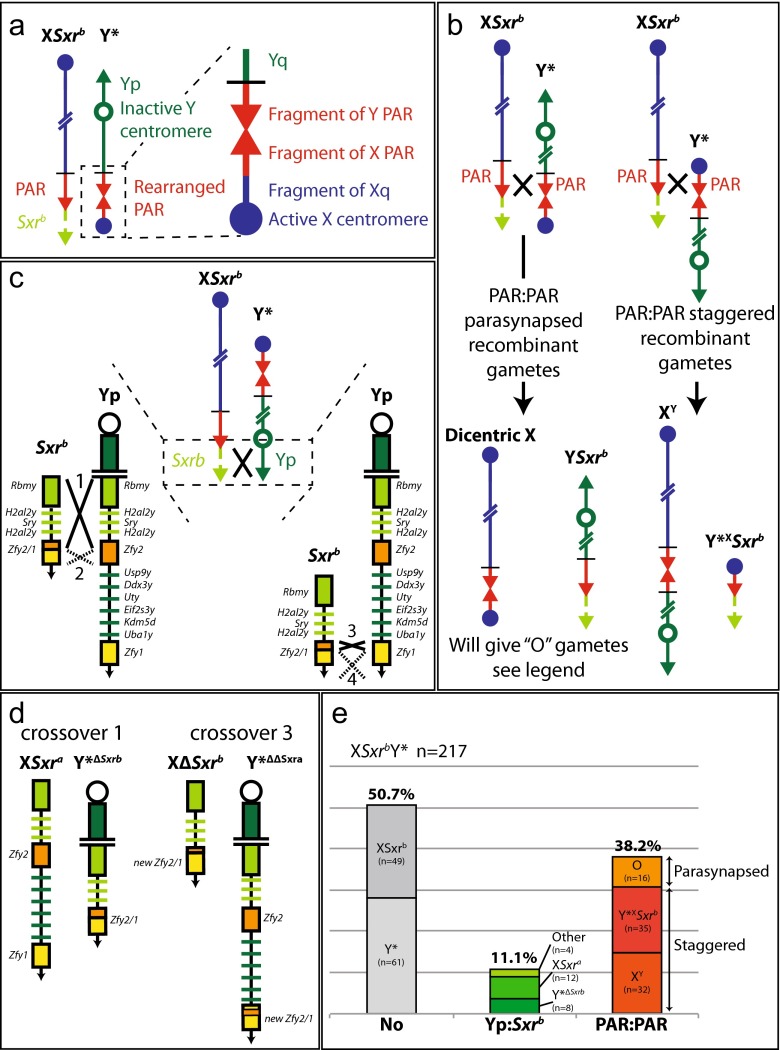
Recombination in X*Sxr* ^*b*^Y* males. (**a**) The sex chromosomes of X*Sxr* ^*b*^Y* males showing the complex Y*PAR and associated X-derived centromere, note that the original Y centromere is inactive. (**b**) PAR-PAR recombination arising from parasynapsed and staggered associations—note that the dicentric X and Y*Sxr* ^*b*^ (with inactive Y centromere) recombinant chromosomes are prone to loss at MI thus generating “O” gametes and are not present in the offspring. (**c**) Yp*-Sxr* ^*b*^ recombination with expanded views showing the Y gene content of the paired segments and potential crossovers—no recombinants were obtained from crossover 2, and recombinants from crossover 4 could not be detected due to a lack of markers. (**d**) The four types of recombinant from crossovers 1 and 3. (**e**) Bar chart of non-recombinant and recombinant frequencies. “Other *n* = 4” comprises one animal from an XΔ*Sxr* ^b^ gamete and three from Y*^ΔΔ*Sxr*a^ gametes (generated by crossover 3)

Mice were produced on an outbred albino MF1 (NIMR stock) background. X*Sxr*^*b*^Y males (Fig. 2a) were generated by mating XY*Sxr*^*b*^ males (*Sxr*^*b*^ attached to the Y PAR) to females carrying the X-autosome translocation T(X;16)16H (T16H/X females). PCR genotyping for *Sxr*^*b*^ markers was then used to identify T16H/X*Sxr*^*b*^ mice that have developed as females–these mice can be female despite the presence of *Sry* in *Sxr*^*b*^, because the presence of the T16H translocation ensures that the inactive X is always the X carrying *Sxr*^*b*^ (Cattanach et al. 1982; McLaren and Monk 1982). The T16H/X*Sxr*^*b*^ females were then mated to XY males, and their X*Sxr*^*b*^Y sons were identified as fertile males (male T16H carriers are sterile) positive for *Sxr*^*b*^ (PCR *Sxr*^*b*^, Table 2). X*Sxr*^*b*^Y* males (Fig. 3a) were originally generated for another study (Vernet et al. 2014). They were produced in the same way as X*Sxr*^*b*^Y males except that the T16H/X*Sxr*^*b*^ females were mated to XY* males (Burgoyne et al. 1998; Eicher et al. 1991).

The X*Sxr*^*b*^Y and X*Sxr*^*b*^Y* males were mated to XX females in order to produce the progeny to be screened for recombinants. In most cases, the females mated to X*Sxr*^*b*^Y* males were homozygous for the X-linked coat marker *Patchy fur* (*Paf*) since this provides an independent check on some of the results obtained from the PCR genotyping (see below). Importantly, it enables the positive identification of XO female progeny as hemizygous *Paf* females, which are known to be produced at an elevated frequency when the Y* chromosome is present (Burgoyne and Evans 2000; Eicher et al. 1991).

### Screening for sex chromosomally recombinant offspring (Tables 1, 2)

**Table 1:** Screening for recombinant offspring from X*Sxr* ^*b*^Y and X*Sxr* ^*b*^Y* matings

X*Sxr* ^*b*^Y	No recombination		*Sxr* ^b^:Yp recombination								PAR:PAR recombination		
			Crossover 1		Crossover 2		Crossover 3		Crossover 4				
Expected gametes	X*Sxr* ^*b*^	Y	X*Sxr* ^*a*^	Y^ΔSxrb^	XΔ *Sxr* ^*a*^	Y^ΔΔ*Sxrb*^	XΔ *Sxr* ^*b*^	Y^ΔΔ*Sxra*^	X*Sxr* ^*b*^	Y	X	Y*Sxr* ^*b*^	
Fur phenotype	Paf/+	Paf	Paf/+	Paf	Paf/+	Paf	Paf/+	Paf	Paf/+	Paf	Paf/+	Paf	
Gender	Male	Male	Male	Male	Male	Male	Male	Male	Male	Male	Female	Male	
Testis size	S	M/L	S	S	S	S	S	M/L	S	M/L		M/L	
PCR *Sxr* ^*b*^	pos	neg	neg	pos	pos	neg	neg	pos	pos	neg		pos	
PCR Yp	neg	pos	pos	neg	pos	neg	neg	pos	neg	pos		pos	
PCR Yq	neg	pos	neg	pos	neg	pos	neg	pos	neg	pos		pos	
PCR X vs Yp	2X noY	1X 1Y	2X 1Y	1X 1Y	2X 1Y	1X noY	1X noY	1X 1Y	2X noY	1X 1Y		1X 1Y	
PCR new *Sxr* ^*b*^							Yes	Yes					
X*Sxr* ^*b*^Y*													
Expected gametes	X*Sxr* ^*b*^	Y*	X*Sxr* ^*a*^	Y*^Δ*Sxrb*^	XΔ *Sxr* ^*a*^	Y*^ΔΔ*Sxrb*^	XΔ *Sxr* ^*b*^	Y*^ΔΔ^ ^*Sxra*^	X*Sxr* ^*b*^	Y*	O	X^Y^	Y* *Sxr* ^*b*^
Fur phenotype	Paf/+	wt	Paf/+	wt	Paf/+	wt	Paf/+	wt	Paf/+	wt	Paf	Paf/+	wt
Gender	Male	Male	Male	Male	Male	Male	Male	Male	Male	Male	Female	Male	Male
Testis size	S	M/L	S	S	S	S	S	M/L	S	M/L			M/L
PCR *Sxr* ^*b*^	pos	neg	neg	pos	pos	neg	neg	pos	pos	neg		neg	pos
PCR Yp	neg	pos	pos	neg	pos	neg	neg	pos	neg	pos		pos	neg
PCR Yq	neg	pos	neg	pos	neg	pos	neg	pos	neg	pos		pos	neg
PCR X vs Yp	2X noY	1X 1Y	2X 1Y	1X 1Y	2X 1Y	1X noY	1X noY	1X 1Y	2X noY	1X 1Y		2X 1Y	1X noY
Y*^X^ qPCR	No Y*^x^								No Y*^x^				Y*^x^
PCR new *Sxr* ^*b*^							Yes	Yes					

*S* small, *M* medium, *L* large, *pos* positive, *neg* negative

**Table 2:** PCR primer sequences, expected amplicon sizes, and annealing temperatures

Primer name	Sequence 5′ to 3′	Test	Amplification in (m = mouse, F = forward, R = reverse)	Expected sizes	Annealing temperature
o3452	GTTAATGAATTAGGGATGGG	PCR *Sxr* ^*b*^	m*Zfy1* and *2* F	852 bp	60 °C
o3072	GTATTAAGTCTTAAAGACATGG		mZfy1 R		
Smc1	TGAAGCTTTTGGCTTTGAG	PCR Yp	m*Kdm5C* and *D* F	330 bp from *Kdm5C* and 300 bp from *Kdm5D*	57 °C
Smc2	CCGCTGCCAAATTCTTTGG		m*Kdm5C* and *D* R		
PC11fp2 F	GTTTTTCCTCAGGTGAGGGA	PCR Yq	m*Ssty* F	237 bp from *Ssty* and 350 bp from *Sstx*	58 °C
PC11fp2 R	CAGAGGGGTCTCTGGAATGT		m*Ssty* R		
Sstxfp10	TCACACAGATAAGAGGGTATTG		m*Sstx* F		58 °C
Sstxrp13	GTTTTCCTATCAGGCCATCCT		m*Sstx* R		
o4110	CAGATCTATGAGGAAGCCAG	PCR X vs Yp	m*Ddx3X* and *Y* F	161 bp from *Ddx3X* and 128 bp from *Ddx3Y*	58 °C
o4111	AAGGACGGACTCTAGATCGG		m*Ddx3X* and *Y* R		
o001	CAAAGTGGGTTTAAGACTGAG	PCR new *Sxr* ^*b*^	m*Zfy1* and *2* F intron 4	166 bp then cut only *Zfy2* with X*ba*I (60 bp + 106 bp)	58 °C
o002	CCAGGAAGTAAGTTCTGAGA		m*Zfy1* and *2* R intron 4		
o003	GGATCTTACTTTTCATTGTTG	PCR new *Sxr* ^*b*^	m*Zfy1* and *2* F intron 9	226 bp then cut only *Zfy2* with S*sp*I (181 bp + 45 bp)	58 °C
o004	GACTGGTACTGTTTGGATTC		m*Zfy 1* and *2* R exon 10		
o005	GAAGATGTTCACTGTTCACA	PCR new *Sxr* ^*b*^	m*Zfy1* and *2* F exon 6	202 bp then cut only *Zfy2* with R*sa*I (118 bp + 84 bp)	58 °C
o006	ACACATGTATAGCTTCACTC		m*Zfy1* and *2* R exon 6		
o021	CTCAGAACCCTTTGGTACAC	PCR new *Sxr* ^*b*^	mZfy1 and 2 F intron 1a	257 bp then cut only *Zfy1* with Sph1 (148 bp + 109 bp)	60 °C
o022	CTTTCCGTTCCCAGAATGCC		m*Zfy1* and *2* R intron 1a		

The initial screen was based on phenotypic markers:

1. *Fur phenotype* (when the mothers were *Paf* homozygotes) A few X*Sxr*^*b*^Y males and nearly all X*Sxr*^*b*^Y* males were mated to females homozygous for the X-linked *Patchy fur* (Paf) mutation (Lane and Davisson 1990). At 10–15 days old, three fur phenotypes are clearly distinguishable (Burgoyne and Evans 2000) that are independent of their Yp/*Sxr* status: wild type (wt) that identifies X^*Paf*^Y* in which the effect of the *Paf* mutation is masked by the presence of the wild type allele on the Y* chromosome, “patchy fur” (Paf/+) due to the very sparse hair in regions where the X^*Paf*^ is expressed as in X^*Paf*^X and X^*Paf*^X^Y^), almost nude (Paf) due to the absence of a paternally derived X as in X^*Paf*^O.
2. *Gender* Based on external examination at weaning.
3. *Testis size* Small 15–26-mg/testis (S) that is associated with the presence of two X chromosomes and/or with the absence of the spermatogonial proliferation gene *Eif2s3y* that is deleted in *Sxr*^*b*^; Medium/Large 70–120-mg/testis (M/L).

PCR analysis using DNA samples was used to confirm and extend diagnoses of sex chromosome complement based on phenotypic markers. They were also used to detect Yp/*Sxr*^*b*^ recombinants based on sequence information for *Sxr*^*b*^ and Yp (Decarpentrie et al. 2012; Mazeyrat et al. 1998; Soh et al. 2014); these were designed to enable the detection of recombination events in homologous regions that would be expected to promote synapsis when juxtaposed during the homology search phase (Supplemental Fig. S1). Primer sequences are in Table 2.

1. *PCR Sxr*^*b*^ (Decarpentrie et al. 2012) The *Zfy2/1* junction fragment was PCR-amplified using primers o3452/o3072. The amplified fragment was then digested using the restriction enzyme R*sa*I generating a 224 bp *Sxr*^*b*^-specific fragment.
2. *PCR Yp* To detect the presence of Yp or *Sxr*^*a*^, we used primers Smc1/Smc2 that amplify a 300-bp fragment of Yp-linked *Kdm5D* (aka *Smcy*) and a 330-bp fragment of X-linked *Kdm5C* (aka *Smcx*) that acts as an amplification control.
3. *PCR Yq* To detect the presence of the long arm of the Y (Yq), we used primers PC11fp2F and R that amplify a 237-bp fragment of *Ssty2* (present in multiple copies) and primers Sstxfp10 and Sstxrp13 that amplify a 350-bp fragment of X-linked *Sstx* (present in multiple copies—Soh et al. 2014) that acts as an amplification control.
4. *PCR X vs Yp* To double-check some genotyping, we performed a qualitative ratio measurement of the X and Yp /Sxr^a^ fragments. Primers o4110 and o4111 (see Table 2) amplify a 161-bp fragment of X-linked *Ddx3x* and a 128-bp fragment of Yp-linked *Ddx3y*. The difference in band intensity gives information about the X vs Yp/*Sxr*^*a*^ dose.
5. *PCR new Sxr*^*b*^ To confirm the products of crossover 3 (XΔ*Sxr*^*b*^ and Y^ΔΔSxra^) that creates a new *Zfy2/1*fusion gene (Supplemental Fig. S1), we have designed several PCR/RFLP tests allowing amplification of both *Zfy1* and *Zfy2* but, thanks to specific SNPs, digestion of only one (primers o001/o002, o003/o004, o005/o006, and o021/o022).
6. Y*^X^*qPCR* (Vernet et al. 2014). To detect the presence of Y*^X^, we utilized qPCRs for X-linked *Prdx4* (absent in Y*^X^), *Amelx* (present in Y*^X^) and *Myog* (on chromosome 1) for normalization.

### Immunostaining to identify X-Y bivalent configurations

In XY males, the frequency of X and Y univalence at pachytene is <5 % but it is usually in excess of 20 % when *Sxr* is present. In an extensive study involving males with *Sxr* attached to the X and/or Y PAR, the X and Y were separated in 26.5–86.9 % of pachytene spermatocytes as compared to 3.5 % in XY controls (Tease and Cattanach 1989). However, in the absence of synapsis, no crossover can form, and the resulting univalence at the first meiotic metaphase (MI) leads to a ∼97 % efficient elimination of the spermatocytes by the MI spindle checkpoint (Burgoyne et al. 1992, 2009; Sutcliffe and Burgoyne 1989; Vernet et al. 2011, 2014). Thus, these spermatocytes with X and Y univalence make a negligible contribution to the offspring. Our interest was therefore restricted to pachytene spermatocytes with X-Y bivalents.

To investigate the X-Y bivalent configurations, we used immunostaining of surface spread spermatocytes as previously described (Turner et al. 2004) using the following antibodies: Guinea pig polyclonal anti-SCP3 (dilution 1:400, gift from James M Turner), mouse monoclonal anti-γH2AX (dilution 1:100, Millipore cat. No. 05–636), Human anti-CREST (dilution 1:50, gift from William Earnshaw). Images were captured on an Olympus IX70 inverted microscope. Each fluorochrome image was captured separately as a 12-bit source image by using a computer-assisted (Deltavision) liquid-cooled CCD (Photometrics CH350L; Sensor: Kodak KAF1400, 1317 × 1035 pixels). A single multiband dichroic mirror was used to eliminate shifts between different filters. Captured images were processed with Fiji and Adobe Photoshop CS5.1.

### Yp:*Sxr*^b^ recombination crossover sizes estimation and location of potential DSB hotspots

Size estimates of Yp, the *Sxr*^*a*^ region, and the sub regions flanking the *Sxr*^*b*^ deletion breakpoints are based on the most recent published sequence of the mouse Y chromosome (Soh et al. 2014—File 3 in Data S1) and the sequence of the *Sxr*^*b*^ deletion breakpoint (Decarpentrie et al. 2012). They are minimum size estimates as the Yp sequence is not yet complete. Three large blocks of N’s within the Yp sequence, totalling 450 kb, were excluded.

The *Sxr*^*a*^ breakpoint has not been sequenced but is known to fall within the distal end of the 37-kb *Rbmy*-repeat tandem array. Based on the recent estimate of 30 *Rbmy* copies in Yp (Soh et al. 2014) and our previous estimate that 1 in 7 *Rbmy*-repeats remain in *Sxr*^*a*^ (Mahadevaiah et al. 1998), we have assumed that the *Sxr*^*a*^ breakpoint lies between the fourth and fifth most distal copies of the *Rbmy*-repeat array.

The Yp estimate was derived from the sequence telomeric to the centromeric heterochromatin, the latter defined as bases 57238–147035 of Genbank AC175459. The Soh et al. sequence includes 10 of the estimated 30 *Rbmy* repeats and we therefore added 740 kb to the Soh et al. sequence length (20 times a repeat unit length of 37 kb).

The Soh et al. sequence does not include the *Prssly* or *Teyorf1* genes, which we have mapped to the *Sxr*^*b*^ region (unpublished data). These two genes are present in an isolated BAC sequence of 185 kb (NW_001034423). We have assumed that *Prssly* and *Teyorf1* are located distal to *Zfy1* since there is no gap in the Soh et al. sequence between *Zfy1* and the *Sxr*^*a*^ breakpoint. We therefore added 185 kb to estimates based on the Soh et al. sequence for Yp and the distance from the telomere to the *Sxr*^*b*^ breakpoint.

Sixteen potential DSB hotspots have been identified in Yp (Brick et al. 2012; Supplementary Data File 1: List of DSB hotspots and H3K4me3 marks). To determine which of these are located within *Sxr*^*b*^, the sequence coordinates were used to identify the associated DNA sequences in the mouse genome using NCBI37/mm9 (as used by Brick et al. 2012), where necessary checking these against the sequence data we have deduced for *Sxr*^*b*^ based on the data from Soh et al. 2014.

## Results and discussion

The recombination data we present here were obtained from PCR genotyping of offspring (see Materials and Methods) that derive from two crosses. Firstly, there are data for 160 offspring obtained from X*Sxr*^*b*^Y × XX matings. Secondly, there are data for 217 offspring from X*Sxr*^*a*^Y* × XX matings.

### Recombination in X*Sxr*^b^Y

As expected, PAR-PAR recombination produces X and Y*Sxr*^*b*^ recombinants (52.5 % of offspring). Yp*-Sxr*^*b*^ recombination produced the recombinants X*Sxr*^a^ and Y^Δ*Sxrb*^ (5.6 %) that are predicted for crossover 1 (Fig. 2a, b, c). Recombinants were not seen for the potential crossover 2 nor were there recombinants deriving from the potential alternative synaptic alignment illustrated in Fig. 3c. Overall, there was a deficiency of non-recombinants (*n* = 67, 41.9 %) as compared to recombinants (*n* = 93, 58.1 %), and this was just significantly different from the expected 50 % of each [Binomial test (http://www.measuringu.com/onep.php); two sided *p* value = 0.0478]. We have no explanation for this deficiency, and it was not observed with the larger sample of data for the X*Sxr*^*b*^Y* model (see below).

### Recombination in X*Sxr*^b^Y*

The Y* chromosome has a complex PAR involving duplication and deletion of PAR DNA sequences and an X-derived centromere distal to this complex PAR (Fig. 3a) (Burgoyne and Evans 2000; Burgoyne et al. 1998; Eicher et al. 1991; Rodriguez and Burgoyne 2001). This leads to two orientations for PAR-PAR synapsis and to the production of four distinct recombinant chromosomes following PAR recombination (Fig. 3b). The opportunities for Yp*-Sxr*^*b*^ synapsis (Fig. 3c) are the same as those in X*Sxr*^*b*^Y. Four types of recombinant arising from crossovers 1 (20 recombinants) and 3 (4 recombinants) were identified (Fig. 3d). The predominance of recombinants involving crossover 1 is to be expected given that it covers 780 kb, which is more than 25-fold longer than for crossovers 2 and 3 (see Table 3). Crossover 4 does cover 580 kb, but we have no markers to detect recombination in this region. The frequencies of the various recombinants identified among the 217 offspring of X*Sxr*^*b*^Y* males are illustrated in Fig. 3e.

**Table 3:** Approximate sizes of PAR, Yp, *Sxr* ^*b*^, and of potential crossover regions

	Areas covered	Homology size regions
Pseudo autosomal region (PAR)		≈700 kb^a^
Short arm of the Y (Yp)	From 30 copies *Rbmy* to distal end of Yp	4 Mb
*Sxr* ^*a*^ domain	From 4 copies *Rbmy* to distal end of Yp	2.24 Mb
*Sxr* ^*b*^ domain	From 4 copies *Rbmy, 2 copies* H2al2y, *Sry*, *Zfy2/1* to distal end of Yp	860 kb
Δ^*Sxrb*^ deletion	From intron 4 *Zfy2* to intron 4 *Zfy1*	1.38 Mb
Yp:*Sxr* ^*b*^ recombination crossover		
Crossover 1	From 5 copies *Rbmy* to *Sxr* ^*b*^ break point	780 kb
Crossover 2	Between *Zfy1* part of *Zfy2/1* and *Zfy2*	29 kb^b^
Crossover 3	Between *Zfy2* part of *Zfy2/1* and *Zfy1*	21 kb^b^
Crossover 4	From *Zfy1* part of *Zfy2/1* to distal end of Yp	580 kb

^a^Perry et al. 2001

^b^Maximum crossover size between *Zfy1* and *Zfy2* with an average of 90 % of homology

How do the recombination data for X*Sxr*^*b*^Y* males compare with those of X*Sxr*^*b*^Y males? As expected, the X*Sxr*^*b*^Y* PAR-PAR recombinants differ due to the presence of the Y* chromosome. There is no deficiency in the number of non-recombinants (*n* = 110, 50.7 %) relative to recombinants (*n* = 107, 49.3 %), but there is an increase in frequency of Yp*-Sxr*^*b*^ relative to PAR-PAR recombinants in X*Sxr*^*b*^Y* (11.1 %, *n* = 24 vs 83) as compared to X*Sxr*^*b*^Y (5.6 %, *n* = 9 vs 84) (*P* = 0.02 Fisher’s exact test, two-tailed). These recombinants include four with exchanges that are generated by crossover 3. The rare recombinants due to crossover 3 were not identified in X*Sxr*^*b*^Y, but this may just reflect the smaller sample size (9 vs 24).

### Is Yp-*Sxr*^b^ recombination potentiated by the attachment of *Sxr*^b^ to the X PAR?

The mouse PAR is estimated to be only ∼700 kb (Perry et al. 2001). Nevertheless, one or two SPO11-mediated DSBs are regularly generated in the X and Y PARs (albeit later than those on autosomes); these DSBs reliably drive the synapsis and formation of a crossover necessary to ensure X and Y segregation at MI (Kauppi et al. 2011, 2012). Yp in normal males is largely protected from recombination because SPO11-mediated DSBs are not preferentially targeted to this region and because it lacks homology with the DSB rich X and Y PARs.

In males with *Sxr*^*b*^ attached to the X PAR, there are two reasons why the Yp-*Sxr*^*b*^ recombination is likely to be preferentially initiated by DSBs in *Sxr*^*b*^. Firstly, we estimate that *Sxr*^*b*^ is 860 kb, whereas Yp we estimate to be 4 Mb; this is due to the 1.38-Mb *Sxr*^*b*^ interstitial deletion (Δ^*Sxrb*^ in Fig. 1d) and the reduced number of copies of *Rbmy* (Mahadevaiah et al. 1998). Thus, 3.14 Mb of Yp has no homology to *Sxr*^*b*^ so that DSBs located in this 3.14 Mb will not be able to find a homologous partner during the presynaptic homology search. On the other hand, with a size of 860 kb, *Sxr*^*b*^ DNA will only very rarely be cut by Spo11 unless this region has one or more DSB hotspots. This led us to hypothesize that *Sxr*^*b*^ may have DSB hotspots because it is attached to the DSB-rich X PAR. Support for this hypothesis is provided by DSB mapping data (Brick et al. 2012). It has been established that the majority of DNA hotspots co-localize with the histone H3 methyl transferase PRDM9, and these authors found that the X PAR together with ∼900 kb of upstream X-specific DNA is unique in having PRDM9-independent DSB hotspots (hotspots that remain in *Prdm9* knockout mice). The frequency of these hotspots declines with increasing distance from the X PAR boundary. We therefore suggest that when *Sxr*^*b*^ is attached to the distal end of the X PAR, it is similarly bestowed with DSB hotspots that decrease in frequency with increasing distance from the PAR. If this is true, the highest density of DSBs should be in the region covered by crossover 1, reducing to a minimum in the region covered by crossover 4. However, if during the homology search, single-stranded DSB tails originating in the region covered by crossovers 3–4 do successfully invade the homologous sequences in Yp, then the resulting synaptic alignment may inhibit exchanges in the region covered by crossovers 1 and 2. We have not identified any double recombinants with PAR-PAR *and Sxr*^*b*^-Yp exchanges; this is presumably a reflection of the low likelihood that both regions of homology are juxtaposed during the presynaptic homology search.

Also of potential relevance to the location of DSBs within *Sxr*^*b*^ are the 16 potential DSB hotspots identified in Yp (Brick et al. 2012; Supplementary Data File 1: List of DSB hotspots and H3K4me3 marks). However, only one (chrY:2158709–2159150) is retained in *Sxr*^*b*^. This is located within each of the few *Rbmy* genes that remain close to the *Sxr*^*b*^ breakpoint that abuts the distal end of the PAR.

### Presynaptic telomere congregation and the incidence of Yp-*Sxr*^b^ recombination

In the 1970s, Solari carried out painstaking electron microscopic reconstructions of the X-Y bivalent at pachytene that served to identify the juxtaposition of what we now know to be the X and Y PAR telomeric ends at the nuclear membrane attachment site and the presence of a synaptonemal complex between the PAR axes; the non PAR ends of the X-Y bivalent although attached to the nuclear membrane were not closely associated (Solari 1970, 1974). The association of the PAR ends is now widely accepted to be initiated prior to synapsis by the congregation of the telomeric ends of all chromosomes during zygotene (bouquet formation), together with dynamic chromosome movements during the bouquet phase that can bring homologous segments in sufficiently close proximity to enable homologous DNA strand invasion and synapsis (Scherthan 2001; Shibuya et al. 2014).

**Fig. 4:**
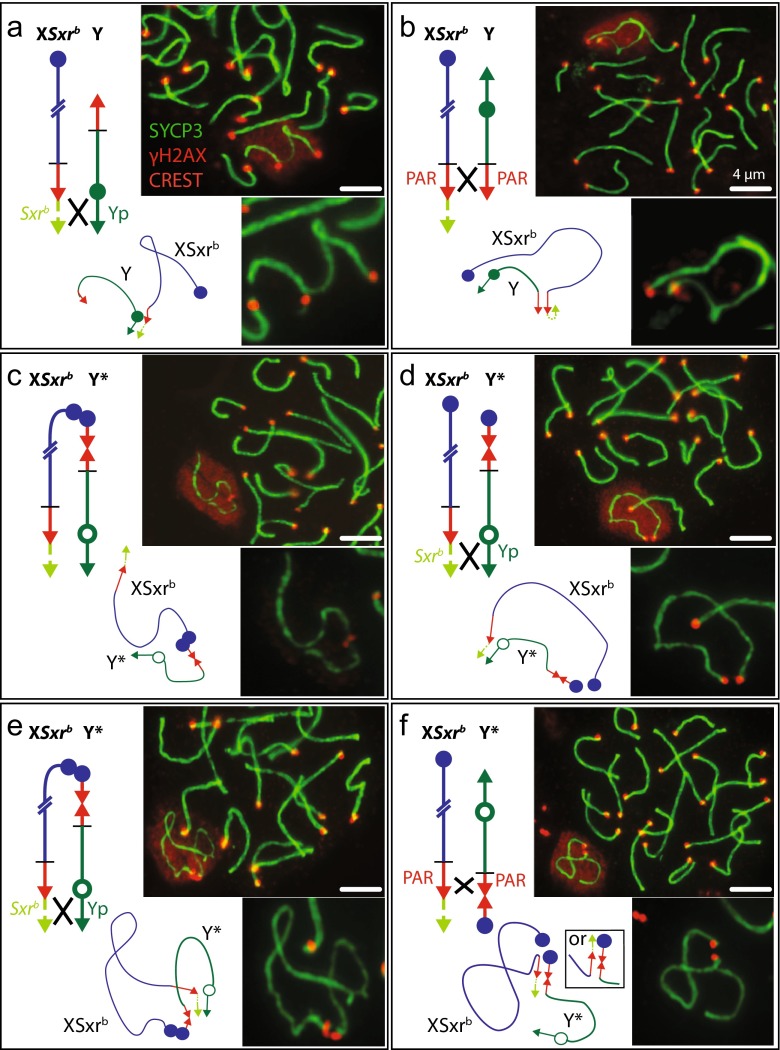
X-Y bivalent configurations at pachytene in X*Sxr* ^*b*^Y and X*Sxr* ^*b*^Y* males. Each panel has a diagram of the predicted crossover event, a low magnification view showing the X-Y bivalent in a γH2AX-stained (*red*) sex chromatin cloud together with near-by autosomes, a higher power view of the X-Y bivalent with the γH2AX staining removed, and a drawing of the deduced X-Y bivalent configuration highlighting the centromere positions. Note that the red CREST staining of active centromeres often appears as *yellow* where it overlaps the green chromosome axis. (**a**) X*Sxr* ^*b*^Y with Yp-*Sxr* ^*b*^ synapsis. (**b**) X*Sxr* ^*b*^Y with PAR-PAR synapsis. (**c**) X*Sxr* ^*b*^Y* with centromere-centromere association—this is likely to be due to synapsis driven by homology of *Sstx* sequences adjacent to the X and Y*(X-derived) centromeres. (**d**) X*Sxr* ^*b*^Y* with Yp-*Sxr* ^*b*^ synapsis. (**e**) X*Sxr* ^*b*^Y* with centromere-centromere *and* Yp-*Sxr* ^*b*^ synapsis. (**f**) X*Sxr* ^*b*^Y* with PAR-PAR synapsis—parasynapsis and staggered synapsis cannot be differentiated at this resolution. The staggered configuration is indicated in the *black square*

How might the telomeric congregation and homology recognition phases be affected in X*Sxr*^*b*^Y and X*Sxr*^*b*^Y* males? In X*Sxr*^*b*^Y the X*Sxr*^*b*^, telomere and adjacent *Sxr*^*b*^ DNA sequences match those of Yp rather than those of Y PAR. This will provide the opportunity for homology recognition and synapsis of *Sxr*^*b*^ with Yp—examples of such bivalents are seen in pachytene spermatocyte spreads (Fig. 4a). Nevertheless, the majority of synaptic associations involve the X PAR/*Sxr*^*b*^ and Y PAR ends (Fig. 4b) and the recombination data document frequent PAR-PAR recombination (Fig. 2c). This is unsurprising since the *Sxr*^*b*^ segment is short (860 kb) so that the X and Y PARs are still close to the membrane attachment sites where the dynamic chromosome movements during the bouquet phase could also lead to PAR-PAR homology recognition.

In X*Sxr*^*b*^Y* the PAR/*Sxr*^*b*^ and Yp, telomeric ends are the same as those of X*Sxr*^*b*^Y thus enabling homology recognition and crossing over. However, there is one feature of Y* that might increase the frequency of Yp*-Sxr*^*b*^ exchanges relative to PAR-PAR exchanges. The PAR end of Y* terminates in an X-derived centromeric region that includes some copies of a multi copy X sequence *DXHXF34* (Laval et al. 1997; Rodriguez and Burgoyne 2001) that derives from a multi copy X gene that we have termed *Sstx* (Touré et al. 2004); recent sequence information from Soh et al. (2014 - see footnote to their Figure 5) has indicated that the majority of copies of *Sstx* are located adjacent to the X centromere. This is expected to promote homology recognition and synapsis of the PAR/centromeric end of Y* with the centromeric end of the X chromosome—examples of this can be seen in pachytene spermatocyte spreads (Fig. 4c). This could lead to crossing over adjacent to the centromeres within the *Sstx* repeat but we have not attempted to detect this. We identified three other pachytene configurations that we interpret as resulting from Yp*-Sxr*^*b*^ synapsis (Fig. 4d), Yp*-Sxr*^*b*^ synapsis and X centromere-Y* PAR/centromere synapsis (Fig. 4e) and PAR-PAR synapsis (Fig. 4f). We therefore wondered whether Y* promotion of alternative synaptic configurations to PAR-PAR synapsis might be responsible for the significant increase in the frequency of Yp*-Sxr*^*b*^ recombinants relative to PAR-PAR recombinants. In order to test this, we classified the synaptic configurations of 99 pachytene spermatocyte spreads from each genotype, which yielded the following results: X*Sxr*^*b*^Y—78 PAR-PAR, 21Yp-*Sxr*^*b*^; X*Sxr*^*b*^Y*—77 PAR-PAR, 18 Yp-*Sxr*^*b*^ (the remaining 4 had the X centromere associated with X-derived centromere of Y* as in Fig. 4c). From this, it is clear that the presence of Y* is not promoting Yp-*Sxr*^*b*^ synapsis at the expense of PAR-PAR synapsis. A plausible alternative explanation is that synapsis of the complex Y* PAR with the X PAR is less likely to result in a crossover because in either the staggered or parasynapsed orientations there is a reduced PAR segment available for homologous synapsis. In those that fail to form a crossover, the X and Y will separate at the end of prophase, be subject to SAC-triggered apoptotic elimination at MI and will thus not contribute to the progeny (Burgoyne et al. 2009; Vernet et al. 2011, 2014).

## Conclusions

The present findings documenting frequent recombination between the Y chromosome short arm (Yp) and the Yp-derived *Sxr*^*b*^ chromosome segment attached distal to the X chromosome PAR, confirm and extend the findings of work published in 1984–1995 (Epplen et al. 1988; Laval et al. 1995; McLaren et al. 1988, 1992; Simpson et al. 1984). Placing these findings in the context of current views as to the transition from meiotic DSB formation through homology search to synapsis has enabled us to propose a model to explain this frequent Yp-*Sxr*^*b*^ recombination. A key issue is the location of the DSBs that generate the single-stranded DNA tail that executes the homology search leading to synapsis between Yp and *Sxr*^*b*^. In normal XY males, DSBs are absent (or extremely rare) at the non-PAR end of the Y. Instead, 1–2 DSBs are directed to the Y PAR; the X PAR also has 1–2 PAR DSBs (Kauppi et al. 2011, 2012). Importantly, DSB mapping has established that there is a spreading of DSB hotspot activity from the X PAR into ∼900 kb of adjacent X-specific DNA (Brick et al. 2012). We now propose that DSB hotspot activity also spreads from the distal end of the X PAR into *Sxr*^*b*^, thus potentiating exchanges with Yp. The next issue is whether there is any likelihood that the single-stranded DNA tails generated within *Sxr*^*b*^ will come close enough to Yp to enable homology recognition and synapsis. We propose that this is likely because the *Sxr*^*b*^ and Yp ends of the chromosomes will be located at nuclear membrane attachment sites, so that telomere congregation and the associated dynamic movement of the clustered chromosome ends during zygotene will promote their interaction (Scherthan 2001; Shibuya et al. 2014). It is very reassuring that the Yp-*Sxr*^*b*^ recombination in these unusual mouse models fits so comfortably with current views as to how homologous synapsis is achieved.

## Electronic supplementary material

Below is the link to the electronic supplementary material.

Supplemental Fig. S1 (a) Diagrams showing 4 potential crossover locations based on regions of homology and (b) the corresponding gametes generated—these were used to plan appropriate PCR assays for genotyping the offspring. Crossover 4 covers the region distal to the *Zfy2/1* breakpoint. Because of a lack of markers we could not identify crossovers in this region, which includes two new genes (*Prssly*, *Teyorf1*) identified by Soh et al (2014) (GIF 81 kb)

High resolution image (EPS 1084 kb)
