# Supplementary figures and images for: Recombination between the mouse Y chromosome short arm and an additional Y short arm-derived chromosomal segment attached distal to the X chromosome PAR
Source: Chromosoma. 2015 Nov 23;125:177–88. doi: 10.1007/s00412-015-0559-0 (PMC4830887; doi:10.1007/s00412-015-0559-0)

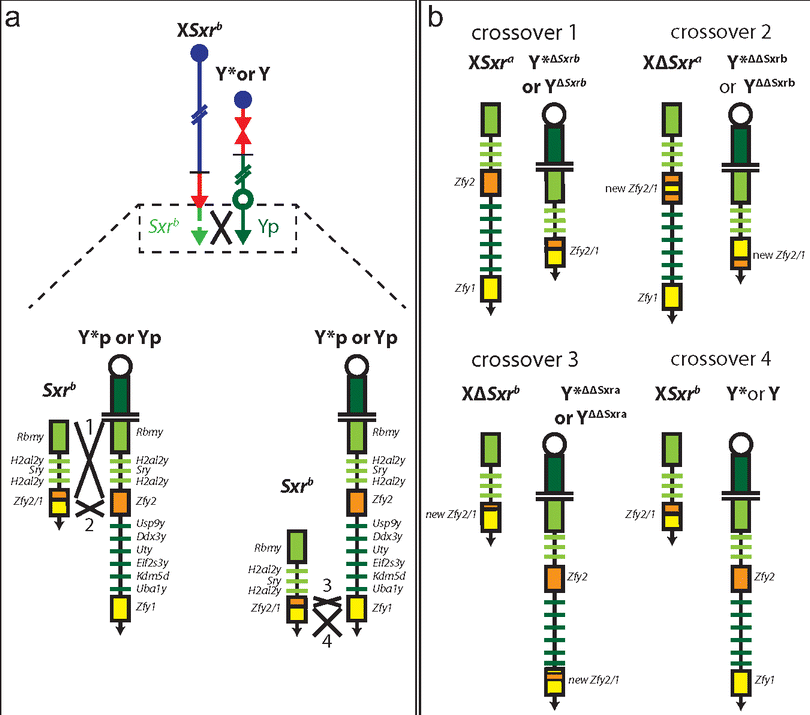

Supplement: Supplementary file 1 — (a) Diagrams showing 4 potential crossover locations based on regions of homology and (b) the corresponding gametes generated—these were used to plan appropriate PCR assays for genotyping the offspring. Crossover 4 covers the region distal to the Zfy2/1 breakpoint. Because of a lack of markers we could not identify crossovers in this region, which includes two new genes (Prssly, Teyorf1) identified by Soh et al (2014) (GIF 81 kb) [file 412_2015_559_Fig5_ESM.gif]
